# Identification of a *De Novo* Deletion by Using A-CGH Involving PLNAX2: An Interesting Candidate Gene in Psychomotor Developmental Delay

**DOI:** 10.3390/medicina58040524

**Published:** 2022-04-08

**Authors:** Noemi Falcone, Annaluisa Ranieri, Andrea Vitale, Lucio Pastore, Barbara Lombardo

**Affiliations:** 1 Department of Molecular Medicine and Medical Biotechnologies, Federico II University, Via Sergio Pansini 5, 80131 Napoli, Italy; falcone@ceinge.unina.it (N.F.); vitalea@ceinge.unina.it (A.V.); lucio.pastore@unina.it (L.P.); 2 CEINGE-Biotecnologie Avanzate, Via G. Salvatore 486, 80145 Naples, Italy; ranieria@ceinge.unina.it

**Keywords:** a-CGH, psychomotor developmental delay, *PLXNA2*

## Abstract

Psychomotor developmental delay is a disorder with a prevalence of 12–18% in the pediatric population, characterized by the non-acquisition of motor, cognitive and communication skills during the child’s development, in relation to chronological age. An appropriate neuropsychomotor evaluation and the use of new technologies, such as Array Comparative Genomic Hybridization (a-CGH) and Next-generation sequencing (NGS), can contribute to early diagnosis and improving the quality of life. In this case, we have analyzed a boy aged 2 years and 8 months, with a diagnosis of psychomotor developmental delay, mainly in the area of communication and language. The a-CGH analysis identified three *de novo* deletions of uncertain clinical significance, involving *PLXNA2* (1q32.2), *PRELID2*, *GRXCR2* and *SH3RF2* (5q32), *RIMS1* (6q13), and a heterozygous duplication of maternal origin involved three genes: *HELZ, PSMD12* and *PITPNC1* (17q24.2). Among all these alterations, our attention focused on the *PLXNA2* gene because of the central function that plexin 2 carries out in the development of the central nervous system. However, all genes detected in the analysis could contribute to the phenotypic characteristics of the patient.

## 1. Introduction

Child development is a gradual and continuous process, with different levels of complexity, with a similar sequence in all children but with a variable rhythm. The different stages of growth include the development of body systems that are responsible for cognitive, physical, and social-emotional skills. Psychomotor developmental delay has a negative impact on social interactions and the performance of daily activities [1]. In fact, psychomotor developmental delay is characterized by the lack of delayed acquisition of motor, cognitive and communication skills during the child’s development, in relation to chronological age. Compared to simple motor retardation, which refers only to global motor skills, psychomotor developmental delay compromises all adaptive functions. The prevalence is 12–18% in developed countries [2,3,4,5,6], where 75% of total cases are genetic and the rest depend on family and social factors [7]. In fact, during their development, children are not passive subjects but actively participate in this process by exploring the environment that surrounds them. In particular, social relationships are fundamental for healthy development, so that the absence of adequate stimulation from the family and social environment could be one of the causes of the delay. Moreover, a delay may be due to the effect of an isolated sensory deficit, such as congenital sensorineural hearing loss or mental deficiency, usually not noticeable until the end of preschool age. Neuropsychomotor evaluation is of fundamental importance to define the level of child development, in order to identify areas of strength and weakness within his development profile and to undertake early rehabilitation treatment [8]. In fact, a study showed that more than 60% of the timely interventions in the psychomotor developmental delay result in a statistically significant improvement [9]. New technologies, such as Array Comparative Genomic Hybridization (a-CGH) and Next-generation sequencing (NGS), can contribute to the identification of variants associated with this delay, thus, representing a great contribution to early diagnosis [10,11,12,13,14,15,16].

## 2. Case Report

**Figure 1:**
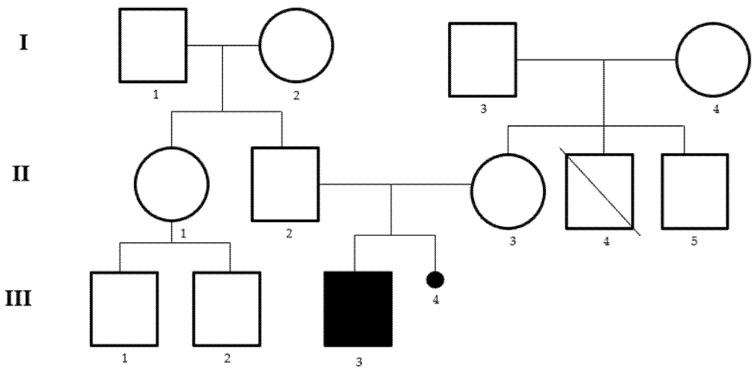
Pedigree of the family. I.2 grandmother ovarian cancer at 29 years, II.3 mother with pituitary microadenoma, II.4 uncle died of acute myocardial infarction at 31 years of age with psychiatric problems, III.3 proband, III.4 miscarriage in a previous pregnancy.

We report the case of a child aged 2 years and 8 months, born from healthy unrelated parents, with a diagnosis of psychomotor developmental delay, mainly in the area of communication and language. The proband was born in the thirty-seventh week of gestation by caesarean section, and the mother reports a threat of abortion in the first trimester and a miscarriage that occurred in a previous pregnancy. The mother was affected by pituitary microadenoma and the grandmother by ovarian cancer at 29 years. The parents report the presence in the family of a maternal uncle with psychiatric problems, who died of acute myocardial infarction (Figure 1). The patient began to take their first steps at the age of 11 months and currently has not yet acquired control of the sphincters and does not say a word. From the age of 18 months, the child has been followed by pediatric neuropsychiatry due to the absence of language and hyperactivity. The electroencephalogram (EEG) reveals brain electrical activity in sleep within normal limits, as well as the ABR (Auditory Brainstem Response) and echo-abdomen test. Physical examination revealed low hairline, wide auricles and feet: clinodactyly of the 4th and 5th toes bilaterally, syndactyly of the 2nd and 3rd toes, large hallux, bilateral flat foot and valgus knees. Furthermore, tone and trophism are normal, and the cranial nerves are unaffected. Currently, the child practices psychomotor and speech therapy twice a week and attends kindergarten without school support and a good interaction with his peers is reported.

**Figure 2:**
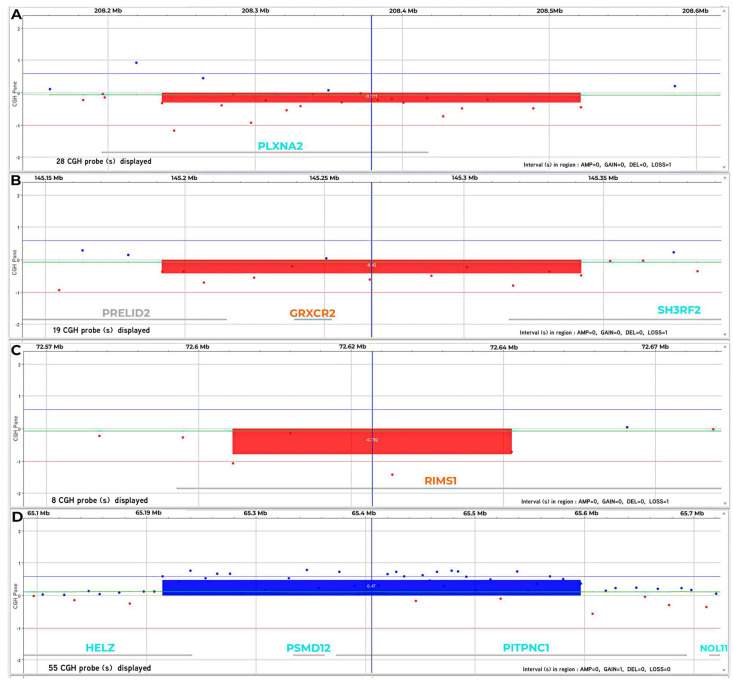
(**A**) a-CGH profile of chromosome 1. This analysis shows a heterozygous *de novo* deletion in 1q32.2 region of 284.57 kb involving *PLXNA2*. (**B**) a-CGH profile of chromosome 5. The analysis shows a heterozygous *de novo* deletion in 5q32 region of 150.23 kb, partially involving *PRELID2*, *GRXCR2* and partially *SH3RF2*. (**C**) a-CGH profile of chromosome 6. This analysis shows a heterozygous *de novo* deletion in 6q13 region of 45.85 kb, involving *RIMS1*. (**D**) a-CGH profile of chromosome 17. The analysis shows a heterozygous duplication of maternal origin in the 17q24.2 region of 381.85 kb partially involving *HELZ*, *PSMD12*, partially *PITPNC1*. Results are interpreted as log2 ratio of test vs. control. The deletion and duplication, when present, is indicated by a rectangle.

Molecular analysis was conducted on DNA of the proband, and his parents extracted this from peripheral blood, with the extraction kit, Maxwell RSC Blood DNA Kit (Promega, Madison, Wisconsin, USA), using the a-CGH platform 4 × 180 K SurePrint G3 Human CGH Microarray (Agilent Technologies, Santa Clara, CA, USA), with an average spacing of 13 kb, allowing an average resolution of 25 kb. The microarray was scanned on an Agilent G2600D scanner. Image files were quantified, and data were visualized by using Agilent’s Cytogenomics software version 4.0.3.12 (Agilent Technologies, Santa Clara, CA, USA). The human assembly utilized was GRCh37, hg19 (http://www.ensembl.org/, last accession 1 April 2022). The a-CGH analysis showed the presence of a heterozygous *de novo* deletion on chromosome 1, in the q32.2 region, ranging from position 208236806 to 208521372, with an extension of 284.57 kb, partially involving the Plexin A2 (*PLXNA2*) (RefSeq # NC_000001.11) gene (Figure 2A), the presence of a heterozygous *de novo* deletion on chromosome 5, in the q32 region, ranging from position 145191813 to 145342040, with an extension of 150.23 kb, partially involving PRELI Domain Containing 2 (*PRELID2*), Glutaredoxin and Cysteine Rich Domain Containing 2 (*GRXCR2*) and partially SH3 Domain Containing Ring Finger 2 (*SH3RF2*) (RefSeq # NC_000005.10) genes (Figure 2B). Furthermore, we also observed the presence of a heterozygous *de novo* deletion on chromosome 6, in the q13 region, ranging from position 72605565 to 72651412, with an extension of 45.85 kb, involving the intron 1 of Regulating Synaptic Membrane Exocytosis 1 (*RIMS1*) (RefSeq # NC_000006.12) gene (Figure 2C) and the presence of a heterozygous duplication of maternal origin on chromosome 17, in the q24.2 region, ranging from position 65214693 to 65596540, with an extension of 381.85 kb, partially involving Helicase With Zinc Finger (*HELZ*), Proteasome 26S Subunit, Non-ATPase 12 (*PSMD12*), partially Phosphatidylinositol Transfer Protein Cytoplasmic 1 (*PITPNC1*) (RefSeq # NC_000017.11) genes (Figure 2D).

## 3. Discussion

The *PLXNA2* gene, located on chromosome 1 cytoband q32.2 (208195588–208417665 (GRCh37/hg19), is characterized by 32 exons, for a total length of about 222,078 bp and mainly expressed in neural tissue. *PLXNA2* belongs to the family of plexin genes, which encode several type 1 transmembrane proteins that function as semaphorin receptors. Semaphorins are plexin ligands and the plexin-semaphorin signaling system is extensively involved in many neuronal events, including driving axons, cell migration, axon pruning and synaptic plasticity [17]. In particular, plexin A2 is thought to bind the secreted proteins semaphorin-3A, -3C, or -5A, thereby precipitating plexin A2 dimerization and the activation of its intrinsic GTPase activating protein domain to act as a negative regulator to Rap1B GTPase. This initiates a signal transduction cascade that mediates axonal repulsion and guidance, dendritic guidance, and neuronal migration during nervous system development [18,19]. Defects in dendritic spine density during development and in adult-born hippocampus [17] and perturbation in migration were shown in a knock-out mouse model for PlxnA2 (PlxnA2^−/−^) of hippocampal granules [20]. Behavioral studies in PlxnA2^−/−^ mice revealed deficits in associative learning, sociability and sensorimotor gating that are common features of neuropsychiatric disorders [18]. All these data support that *PlxnA2* expression is relevant to the structure and function of the nervous system. For this reason, *PlxnA2* dysfunction appears to contribute to the development of neurological disorders, such as intellectual disability (ID), retardation and schizophrenia [19]. Differences in phenotypes may be explained by the incomplete penetrance of the gene. Only in very rare cases, mutations in the *PLXNA2* gene can be associated with cardiac abnormalities [21]. The gene is not present in the Simons Foundation Autism Research Initiative (SFARI) database (https://gene.sfari.org/, last accession 1 April 2022), unlike the other genes in the same family, *PLXNA3* and *PLXNA4*, which appear to be closely related to ASD. Indeed, a *de novo* variant of the junction site in the *PLXNA3* gene was identified in a proband with ASD [22] and targeted sequencing of 3195 Chinese probands [23] identified three rare deleterious variants in *PLXNA3* in Chinese ASD probands. To date, most of the studies on the *PLXNA2* gene identify single nucleotide polymorphisms (SNPs) and missense point mutations that contribute to altering the function of the protein and, thus, contribute to candidate *PLXNA2* for the development of intellectual disability.

With the implementation of techniques, such as a-CGH and NGS, it has been observed that chromosomal aberrations are found in the genome of 15% of patients with ID, while in 36% of cases, the disorder is related to single-gene variations. Finally, single-gene variations are *de novo*, while in only 4% of cases, they are inherited in an autosomic recessive manner [19]. In this case, a *de novo* deletion was identified in the heterozygosity of the *PLXNA2* gene, extending from exon 1 to exon 12, partially including intron 12. This deletion is reported of uncertain clinical significance on the consulted databases Clinvar (https://www.ncbi.nlm.nih.gov/clinvar/, accessed on 1 April 2022), Decipher (https://decipher.sanger.ac.uk/, accessed on 1 April 2022), Database of Genomic Variants (http://dgv.tcag.ca/dgv/app/home, accessed on 1 April 2022), GeneCards (http://www.genecards.org/, accessed on 1 April 2022), and OMIM (https://www.omim.org/, accessed on 1 April 2022). The alteration involves the binding site of plexin with semaphorin and the absence of this site alters the entire cellular pathway, in which the *PLXNA2* gene is involved. In light of the observations in the literature, we can hypothesize a possible role of the altered *PLXNA2* gene in the development of phenotypic characteristics detected in the proband and in other patients with dysmorphism and cardiac anomalies. Further studies are needed to elucidate the role of the gene in the pathogenesis of psychomotor developmental delay.

In addition, we found a *de novo* heterozygous deletion that includes the *GRXCR2* and *SH3RF2* genes located on chromosome 5, cytoband q32. The *GRXCR2* gene (145239296–145252531 (GRCh37/hg19) consists of three exons, for a total length of about 13,236 bp and encodes a protein, which causes the loss of hearing. *GRXCR2* and its paralogue *GRXCR1* have a size of ~30 kDa and are highly conserved cytosolic proteins. The mutation of *GRXCR2* has been associated with autosomal recessive non-syndromic deafness [24], characterized by moderate to severe bilateral hearing loss. In particular, *GRXCR2* is concentrated in the basal region of the stereocilia and is essential for the localization of taperin. In Grxcr2-deficient hair cells, taperin is reduced at the base and is found along the length of the stereocilia, which are elongated and disorganized; therefore, *GRXCR2* would seem to limit the taperin at the base of the stereocilia, causing deafness [25,26].

The SH3 protein, encoded by the *SH3RF2* gene, is an E3 ubiquitin-protein ligase [27]. It acts as an anti-apoptotic regulator of the JNK pathway by ubiquitinating and promoting the degradation of SH3RF1, a scaffold protein required for the pro-apoptotic activation of JNK [28]. Studies using *SH3RF2* knockout mice show that haploinsufficiency of the gene is related to unilateral disorders of hippocampal function and autistic behaviors, social and communication interactions, repetitive behaviors and seizures [29].

Finally, a maternal duplication involves the *PSMD12* gene. The *PSMD12* gene is located on chromosome 17 of the cytoband q24.2 (65336619–65362721 (GRCh37/hg19), for a length of about 26.103 bp and 11 component exons of the proteasome regulatory subunit, a large multi-subunit enzyme complex responsible for ATP-dependent and ubiquitin-mediated degradation of proteins and is highly conserved. In particular, *PSMD12* is required for proper assembly and localization of the proteasome [30] and is highly expressed in the brain. Recently, *de novo* mutations in *PSMD12* have been identified in individuals with ID and ASD [31].

## 4. Conclusions

The a-CGH technique represents an important advance in the screening of unbalanced rearrangements, caused by the loss and/or duplication of small genomic material, not detectable by other cytogenetic methods, acting as a fundamental diagnostic tool in the identification of the molecular component underlying multiple genotype–phenotype relationships [32,33,34,35,36]. Due the significant increase in resolution, it is possible to identify the presence of causative or potentially causative chromosomal abnormalities on the whole genome, in a great variety of cases, from the most common neurological and congenital disorders to multifactorial syndromes, with advantages in terms of time, precision and standardization [37,38,39].

In this study, the a-CGH detected copy number variations (CNVs) containing genes potentially related to the clinical characteristics of the patient. In particular, the involvement of the *PLXNA2* gene in the structure and function of the central nervous system and its implication in neurological disorders make it an interesting candidate gene for neurodevelopmental disorders. Furthermore, the other alterations identified in the patient could contribute to the severity of the phenotype, with roles not yet defined.

For this reason, the progressive evolution of the a-CGH, through the use of higher resolution platforms, such as the SNP-array technology, may lead, in the near future, to its increasingly massive use for the benefit of understanding the disease’s origin of still unknown etiology. Finally, a combination between the a-CGH and NGS could represent an added value to the diagnosis, providing a complete diagnosis of the patients and helping to provide further information on the causal mechanisms involved in neurodevelopmental disorders and congenital anomalies.

## Data Availability

The data will be available by contacting the corresponding authors.
